# Correlation study between inflammatory, nutritional and metabolic indicators and the risk of invasive ductal carcinoma of the breast

**DOI:** 10.3389/fonc.2026.1752259

**Published:** 2026-07-31

**Authors:** Peilin Chen, Xinning Lv, Hang Su, Hongfeng Wu, Ziyao Zhang, Bingjie Zhang, Fenghua Zhang

**Affiliations:** 1 Graduate School, Hebei Medical University, Shijiazhuang, China; 2 Department of Breast and Thyroid Surgery, Hebei General Hospital, Shijiazhuang, China; 3 Department of Gynecology, Hebei General Hospital, Shijiazhuang, China; 4 General Affairs Office, Hebei General Hospital, Shijiazhuang, China; 5 Department of Breast Surgery IV, Affiliated Hospital of Hebei University of Engineering, Handan, China

**Keywords:** invasive breast cancer, logistic regression analysis, metabolic syndrome, nutritional prognosis index, risk of disease onset, systemic inflammatory response index

## Abstract

**Background:**

The aim of this study was to compare clinical data between women with invasive breast cancer (IBC) and those with benign breast tumors, identify risk factors influencing IBC development, and provide evidence for early clinical identification and intervention.

**Methods:**

Clinical records of 1, 049 female patients with breast masses who underwent surgical treatment at Hebei Provincial People’s Hospital between October 1, 2018, and October 1, 2020, were retrospectively collected. The cohort comprised 738 cases in the benign breast mass group and 311 cases in the invasive breast cancer group. Chi-square (χ²), Z-tests, and T-tests were used to compare the two groups regarding age, menopausal status, marital status, parity, family history of breast cancer, history of benign breast surgery, presence of metabolic syndrome (MS), and related inflammatory and nutritional indicators [prognostic nutritional index (PNI), albumin/globulin ratio (AGR), systemic inflammatory response index (SIRI), platelet/lymphocyte ratio (PLR), neutrophil/monocyte ratio (NMR). Independent risk factors for female IBC development were identified through univariate and multivariate logistic regression analyses.

**Results:**

Logistic Regression Analysis: Univariate analysis revealed that age, postmenopausal status, marital history, parity, coexisting multiple sclerosis, PNI, AGR, SIRI, and history of benign breast surgery were associated with inflammatory breast cancer in women. Multivariate analysis indicated that age, postmenopausal status, coexisting multiple sclerosis, decreased PNI, elevated SIRI, and history of benign breast surgery were independent risk factors for inflammatory breast cancer in women.

**Conclusions:**

Advanced age, postmenopausal status, concomitant metabolic syndrome, decreased PNI, and elevated SIRI are independent risk factors for invasive breast cancer in women. A history of benign breast surgery may serve as a protective factor. Clinicians should enhance screening and intervention for high-risk populations based on these factors.

## Introduction

Invasive breast cancer (IBC) is a leading cause of cancer incidence and mortality among women worldwide. In 2020, there were 2.3 million new cases and 685, 000 deaths globally, accounting for one-quarter of all female cancer cases and one-sixth of all female cancer deaths, posing a serious threat to women’s health (1). Research indicates that approximately 30% of global breast cancer cases are associated with modifiable risk factors such as overweight and physical inactivity (2). Therefore, identifying independent risk factors for IBC onset is crucial for screening high-risk populations, enabling early intervention, and improving patient prognosis. Inflammation, nutritional status, and metabolic abnormalities play pivotal roles in tumorigenesis and progression (3). Inflammatory markers derived from complete blood counts have demonstrated predictive value in cancers like colorectal cancer (4), but current research on their association with breast cancer primarily focuses on prognosis rather than disease prediction (5). Although indicators such as metabolic syndrome (MetS) and the Prognosis Nutrition Index (PNI) have been shown to correlate with breast cancer risk, the underlying mechanisms remain incompletely understood (6), and studies incorporating multiple indicators into a combined risk assessment system are lacking.

The aim of this study was to investigate the correlations between inflammatory, nutritional, and metabolic indicators and the risk of invasive breast cancer (IBC), identify independent risk factors for IBC development, and provide evidence for developing personalized clinical prevention and control strategies.

## Materials and methods

### Patients

This study included 1, 486 patients who underwent breast mass excision at the Department of Breast and Thyroid Surgery, Hebei General Hospital between October 2018 and October 2020.

For this study, we enrolled female patients aged 18 years or older who had received definitive diagnoses of either benign breast tumors or invasive breast cancer (IBC) via postoperative paraffin-embedded tissue pathology. All included patients had complete medical records covering general demographic characteristics, medical history, preoperative laboratory test results, and final pathological reports, and none had a prior history of other malignancies.

Patients were excluded from the analysis if they were male, had pathological diagnoses of non-invasive breast cancer or recurrent breast cancer, were suffering from concurrent malignancies, or had incomplete clinical data (e.g., missing key laboratory indicators or fragmented medical history records).

### Data collection and follow-up

Demographic characteristics, along with relevant test and examination results upon admission, were retrieved from the hospital medical record management system. These included age, menopausal status, marital status, parity, family history of breast cancer, history of benign breast surgery, presence of metabolic syndrome.

We calculated five inflammation and nutrition-related biomarkers using preoperative blood test results: (1) Prognostic Nutritional Index (PNI), calculated as 10 × serum albumin (g/dL) + 0.005 × peripheral blood lymphocyte count (/mm³); (2) Albumin/Globulin Ratio (AGR), defined as the ratio of serum albumin to serum globulin; (3) Systemic Inflammatory Response Index (SIRI), calculated as (neutrophil count × monocyte count)/lymphocyte count (all units: ×10^9^/L); (4) Platelet/Lymphocyte Ratio (PLR), the ratio of platelet count to lymphocyte count; and (5) Neutrophil/Monocyte Ratio (NMR), the ratio of neutrophil count to monocyte count. All blood samples for these assays were collected and analyzed within 7 days before surgery.

### Statistical analysis

Data analysis was performed using SPSS 26.0 software. For quantitative data, normally distributed variables were expressed as x ± s and compared between groups using t-tests; non-normally distributed variables were expressed as M (Q1, Q3) and compared between groups using Z-tests. Count data were expressed as counts (percentages) [n (%)], with intergroup comparisons performed using the χ² test. Univariate logistic regression analysis was used to screen factors associated with female IBC incidence. Variables with P<0.05 in univariate analysis were included in multivariate logistic regression analysis to identify independent risk factors. P<0.05 was considered statistically significant.

### Ethical approval

This study was carried out in strict accordance with the principles of the Declaration of Helsinki. The retrospective study was approved by the Ethics Committee of Hebei General Hospital and patients’ informed consent was exempted(2025-LW-0242).

## Results

### Comparison of clinical data differences

Among the 1, 049 enrolled patients, 738 were classified as benign breast lesions and 311 as IBC. Significant intergroup differences were observed in key clinical and laboratory indicators, including age, postmenopausal status, history of benign breast surgery, presence of metabolic syndrome (MS), and the inflammatory/nutritional markers PNI, AGR and SIRI (all P<0.05). Marital status and parity also showed marginal differences between groups (P<0.05). No statistically significant associations were found between IBC status and family history of breast cancer, PLR or NMR (all P>0.05). Detailed results are presented in **Table 1**.

**Table 1:** Comparison of clinical data differences.

Clinical indicators	All patients (n=1049) n(%)/IQR/M ± SD	Benign breast tumor group (n=738) n(%)/IQR/M ± SD	IBC group (n=311) n(%)/IQR/M ± SD	χ^2^/Z/t	P
Age(years)	45 (34, 54)	40(30, 48)	55(47, 64)	-16.528	<0.001
Menopausal Status Premenopausal Postmenopausal	743 (70.8) 306 (29.2)	619 (83.9) 119 (16.1)	124 (39.9) 187 (60.1)	205.053	<0.001
Marital Status Unmarried Married Divorced or Widowed	108 (10.3) 926 (88.3) 15 (1.4)	105 (14.2) 628 (85.1) 5 (0.7)	3 (1.0) 298 (95.8) 10 (3.2)	50.090	<0.001
Parity Status Nulliparous 1-2 Childbirths ≥3 Childbirths	148 (14.1) 827 (78.8) 74 (7.1)	141 (19.1) 565(76.6) 32(4.3)	7(2.3) 262(84.2) 42(13.5)	71.770	<0.001
Family History of Breast Cancer Negative Positive	1038(99.0) 11(1.0)	733(99.3) 5(0.7)	305(98.1) 6(1.9)	–	0.094
History of Benign Breast Surgery Negative Positive	1001(95.4) 48(4.6)	696(94.3) 42(5.7)	305(98.1) 6(1.9)	7.091	0.008
MS Negative Positive	923(88.0) 126(12.0)	689(93.4) 49(6.6)	234(75.2) 77(24.8)	67.968	<0.001
PNI	52.19 ± 3.85	52.74 ± 3.67	50.89 ± 3.97	7.257	<0.001
AGR	1.55 (1.40, 1.73)	1.58 (1.43, 1.76)	1.49 (1.35, 1.64)	-5.431	<0.001
SIRI	0.52 (0.36, 0.75)	0.49 (0.34, 0.70)	0.56 (0.40, 0.86)	-4.283	<0.001
PLR	157.14 (123.95, 196.55)	155.95 (122.26, 196.05)	158.97 (126.92, 201.47)	-1.158	0.247
NMR	14.03 (11.32, 17.28)	13.86 (11.27, 17.15)	14.20 (11.50, 17.76)	-1.190	0.234

PNI, Prognostic Nutritional Index; AGR, Albumin/Globulin Ratio; SIRI, Systemic Inflammatory Response Index; PLR, Platelet/Lymphocyte Ratio; NMR, Neutrophil/Monocyte Ratio; MS, Metabolic Syndrome; IQR, interquartile range; M ± SD, Mean ± Standard Deviation.

### Univariate logistic regression analysis of IBC risk in women

We performed univariate logistic regression analysis using IBC status as the dependent variable (0 = benign breast tumor, 1 = IBC), with all indicators from **Table 1** included as independent variables. The results showed that age, postmenopausal status, marital status (married, divorced or widowed), parity (1–2 births, ≥3 births), history of benign breast surgery, presence of metabolic syndrome (MS), PNI, AGR and SIRI were significantly associated with the risk of female IBC (all P < 0.05). In contrast, family history of breast cancer, PLR and NMR showed no significant association with IBC risk (all P > 0.05). Detailed results are presented in **Table 2**.

**Table 2:** Univariate logistic regression analysis of IBC risk in women.

Medical record data	*B*	*OR*	95%*CI*	*P*
Age	0.109	1.115	1.009~1.132	<0.001
Menopausal Status Premenopausal Postmenopausal	2.060	7.844	5.811~10.589	Ref. <0.001
Marital Status Unmarried Married’ Divorced or Widowed	2.826 3.076	16.871 21.667	5.310~53.603 5.674~82.743	Ref. <0.001 <0.001
Reproductive History Nulliparous 1-2 Childbirths ≥3 Childbirths	2.272 2.770	9.701 15.962	4.478~21.018 6.753~37.729	Ref. <0.001 <0.001
Family History of Breast Cancer Negative Positive	1.059	2.884	0.874~9.521	Ref. 0.082
History of Benign Breast Surgery Negative Positive	-1.121	0.326	0.137~0.775	Ref. 0.011
MS Negative Positive	1.532	4.627	3.140~6.818	Ref. <0.001
PNI	-0.132	0.876	0.844~0.910	<0.001
AGR	-1.421	0.241	0.140~0.417	<0.001
SIRI	0.644	1.904	1.388~2.611	<0.001
PLR	0.001	1.001	0.999~1.003	0.246
NMR	0.021	1.021	0.997~1.045	0.082

PNI, Prognostic Nutritional Index; AGR, Albumin/Globulin Ratio; SIRI, Systemic Inflammatory Response Index; PLR, Platelet/Lymphocyte Ratio; NMR, Neutrophil/Monocyte Ratio; MS, Metabolic Syndrome.

### Multivariate logistic regression analysis for female IBC risk

Variables with P<0.05 in univariate analysis (age, menopausal status, marital status, parity, history of benign breast surgery, presence of MS, PNI, AGR, SIRI) were included in the multivariate logistic regression analysis. Results showed: age (OR = 1.102, 95% CI: 1.077–1.128, P<0.001), postmenopausal status (OR = 1.763, 95% CI: 1.127–2.760, P = 0.013), MS comorbidity (OR = 1.878, 95% CI: 1.175–3.000, P = 0.008), Decreased PNI (OR = 0.938, 95% CI: 0.895–0.983, P = 0.008), elevated SIRI (OR = 1.906, 95% CI: 1.265–2.871, P = 0.002), and history of benign breast surgery (OR = 0.322, 95% CI: 0.127–0.813, P = 0.016) were independent risk factors for female IBC. Marital status, parity, and AGR did not enter the final model (all P>0.05). Detailed results are shown in **Table 3**.

**Table 3:** Multivariate logistic regression analysis for female IBC risk.

Medical record data	*B*	*OR*	95%*CI*	*P*
Age	0.097	1.102	1.077~1.128	<0.001
Menopausal Status Premenopausal Postmenopausal	0.567	1.763	1.127~2.760	Ref. 0.013
Marital Status Unmarried Married Divorced or Widowed	0.057 -1.186	1.058 0.305	0.173~6.487 0.40~2.321	Ref. 0.951 0.252
Reproductive History Nulliparous 1-2 Childbirths ≥3 Childbirths	0.309 -0.582	1.362 0.559	0.400~4.641 0.146~2.143	Ref. 0.621 0.396
History of Benign Breast Surgery Negative Positive	-1.134	0.322	0.127~0.813	Ref. 0.016
MS Negative Positive	0.630	1.878	1.175~3.000	Ref. 0.008
PNI	-0.064	0.938	0.895~0.983	0.008
AGR	-0.492	0.611	0.317~1.178	0.142
SIRI	0.645	1.906	1.265~2.871	0.002

PNI, Prognostic Nutritional Index; AGR, Albumin/Globulin Ratio; SIRI, Systemic Inflammatory Response Index; MS, Metabolic Syndrome.

## Discussion

Studies have shown that inflammation is involved in all stages of tumor initiation, progression and invasion and impacts patient prognosis (1) Cancer cells interact with surrounding stromal cells and inflammatory cells to form an inflammatory tumor microenvironment that promotes tumor growth. Complete blood count (CBC) is a routine test for breast cancer patients, offering the advantages of simplicity, cost-effectiveness and reproducibility, and is more practical than imaging studies, pathological examinations and other blood biomarkers. Peripheral blood inflammatory markers can partially reflect the systemic inflammatory state of the body (7) and have demonstrated predictive value in cancers such as colorectal cancer (4). However, current studies primarily focus on their prognostic significance in breast cancer, with very limited exploration of their predictive role in disease onset.

Concurrently, patients with metabolic syndrome or malnutrition have higher cancer susceptibility, increased recurrence rates and poorer long-term prognosis, and these conditions have been identified as potential risk factors for pancreatic, breast, prostate and colorectal cancers (8). Nevertheless, as the association between these factors and breast cancer pathogenesis remains unproven (6), whether primary prevention of breast cancer targeting metabolic and nutritional factors is warranted remains controversial. In summary, integrating nutritional, metabolic and inflammatory markers into the invasive breast cancer (IBC) risk assessment system to identify independent risk factors provides new perspectives for studying the incidence risk of IBC. In this study, we conducted a large retrospective cohort analysis, incorporating demographic information and clinical laboratory data, to identify high-risk factors for IBC development among inflammatory, nutritional and metabolic indicators. Descriptive statistics and univariate/multivariate logistic regression analyses confirmed that age, menopause status, history of benign breast surgery, prognostic nutritional index (PNI), coexisting metabolic syndrome (MetS) and systemic inflammation response index (SIRI) are independent risk factors for IBC.

Adverse lifestyles (e.g., high-fat diet, physical inactivity) readily induce metabolic syndrome (MetS), which plays a pivotal role in breast cancer development and progression. A meta-analysis of 392, 583 women (19, 628 breast cancer cases) showed that MetS increased overall breast cancer risk by 52% (RR = 1.49, p<0.0001), with an even higher risk in postmenopausal women (RR = 2.01, p<0.001) (9). Notably, breast cancer risk rose stepwise with the number of MetS components: no significant change with 1 component (RR = 1.00, p=0.976), slight increase with 2 components (RR = 1.40, p=0.121), and a significant 1.98-fold increase with ≥3 components (p<0.001) (9). Another meta-analysis of 602, 195 women (15, 945 cases) confirmed this association (adjusted RR = 1.15, p=0.003), again stronger in postmenopausal women (adjusted RR = 1.25, p<0.001) (6). Our findings further validated this link: MetS patients had an 87% higher breast cancer risk (OR = 1.878, P = 0.008). The primary underlying mechanism is obesity-induced systemic estrogen elevation: in postmenopausal women, adipose tissue becomes the main source of estrogen via aromatase-mediated conversion of adrenal androgens to estrone and estradiol (10), which enhances estrogen receptor signaling and drives breast epithelial proliferation and tumorigenesis.

Insulin resistance (IR), another core feature of metabolic syndrome, has been independently associated with an increased risk of breast cancer. IR is typically an acquired condition defined as reduced physiological responsiveness of target tissues (e.g., liver, muscle, adipose tissue) to insulin stimulation (11), which impairs glucose disposal and leads to increased insulin synthesis by pancreatic β-cells and subsequent hyperinsulinemia. The core carcinogenic mechanism lies in the bidirectional crosstalk between estrogen signaling and the insulin/insulin-like growth factor-1 (IGF-1) axis: IGF-1 is a peptide hormone structurally homologous to insulin with potent anabolic and mitogenic effects (12). Insulin and IGF-1 can modulate estrogen receptor alpha (ERα) activity through both genomic and non-genomic mechanisms, including phosphorylation of ERα and its co-regulators via the downstream PI3K/AKT/mTOR and Ras/Raf/MEK/ERK signaling cascades (13, 14). Specifically, the PI3K/AKT/mTOR axis promotes protein synthesis, cell proliferation and metabolic reprogramming while inhibiting apoptosis by phosphorylating pro-apoptotic proteins and activating anti-apoptotic members of the Bcl-2 family. Meanwhile, the Ras/Raf/MEK/ERK signaling pathway drives cell cycle progression and enhances mitotic activity, both of which collectively promote tumorigenesis and progression. Therefore, sustained activation of these pathways by hyperinsulinemia can significantly accelerate the carcinogenic process.

Adipose tissue secretes multiple bioactive adipokines that exert systemic effects on metabolism, immune function and tumor biology (15). Leptin, a key adipokine, is consistently implicated in breast cancer progression: hyperleptinemia (prevalent in metabolic syndrome) promotes cell proliferation, angiogenesis and anti-apoptosis by activating JAK/STAT, PI3K/AKT and MAPK signaling pathways (16). In contrast, adiponectin exhibits antiproliferative, insulin-sensitizing and anti-inflammatory properties; it promotes APPL-1/AMPK interaction, and AMPK inhibits multiple oncogenic pathways involved in cell cycle initiation, growth and survival, including ERK1/2, PI3K/Akt, cJNK and STAT3 (16). The high-leptin/low-adiponectin imbalance characteristic of insulin resistance and MetS thus drives breast cancer development. Additionally, obesity induces chronic low-grade inflammation in adipose tissue: obese adipocytes secrete proinflammatory cytokines such as TNF-α and IL-6, which activate the “obesity-inflammation-aromatase axis” (17). Collectively, MetS regulates breast cancer development through a multidimensional signaling network involving endocrine disruption, metabolic abnormalities and chronic inflammation, with four interconnected core mechanisms: abnormal estrogen metabolism, insulin resistance, adipokine imbalance and chronic inflammation, which synergistically promote carcinogenesis.

The Prognostic Nutritional Index (PNI), calculated from routine serum albumin and lymphocyte count, is a well-established biomarker for evaluating systemic inflammation, nutritional status and immune function. Its association with the risk and prognosis of various malignancies has been extensively documented, but research on its specific role in breast cancer development remains limited. The core finding of this study is that reduced PNI (OR = 0.938, P = 0.008) is a protective factor against invasive breast cancer (IBC), meaning each 1-unit increase in PNI corresponds to a 7.2% lower breast cancer risk. This finding is highly consistent with the 2025 study by Rong Z et al., which reported a 4% risk reduction per 1-unit PNI increase (fully adjusted OR = 0.96; 95% CI: 0.94-0.98) (18), further validating the negative correlation between PNI and breast cancer risk.

PNI’s core value lies in quantifying the synergistic homeostasis between the body’s nutritional reserves and immune function. Its association with breast cancer development manifests primarily through two dimensions: nutritional reserve imbalance and immune dysfunction. Decreased serum albumin indicates a hypercatabolic state, accelerating muscle protein breakdown and inducing the Warburg effect in the tumor microenvironment. The metabolic byproduct lactate acidifies the microenvironment, directly suppressing immune cell activity and facilitating cancer immune evasion (19). Meanwhile, PNI modulates antitumor immunity by affecting lymphocyte function: the high PNI group shows significantly higher lymphocyte and fibroblast infiltration in the tumor microenvironment, indicating stronger antitumor immune responses (20). Choi et al. further elucidated that prolonged malnutrition inhibits bone marrow hematopoiesis and reduces lymphocyte production, while tumor-associated inflammation accelerates lymphocyte apoptosis, creating a “reduced production and increased consumption” dilemma (21). Indirect corroboration comes from colorectal cancer studies, where low PNI patients have lower preoperative lymphocyte counts and delayed postoperative immune recovery, impairing the clearance of tumor micrometastases.

Regarding the specific mechanisms of immune function impairment: reduced serum albumin levels associated with low PNI directly impede the formation of immune synapses—the critical structure enabling T cells to specifically recognize cancer cells, preventing effective T cell-tumor cell binding. Meanwhile, the malnutrition-induced immunosuppressive microenvironment upregulates immune exhaustion markers such as PD-1 and TIM3 on CD8^+^ T cells, completely abolishing their tumoricidal activity and further dismantling the body’s anti-tumor immune defenses.

PNI is not only closely associated with breast cancer risk but also has significant prognostic value. Chen et al. (22) retrospectively analyzed 785 breast cancer patients and identified 51 as the optimal PNI cutoff for prognosis prediction. Results showed that patients with higher PNI levels had significantly prolonged disease-free survival (DFS) and overall survival (OS) (P<0.001), highlighting PNI’s potential as a clinical assessment indicator and providing evidence for its integration into comprehensive breast cancer diagnosis and treatment systems.

Systemic inflammation plays a crucial role in the progression and treatment outcomes of various cancers including breast cancer. The systemic inflammation response index (SIRI), a valuable hematological biomarker derived from routine blood tests, reflects the balance between pro-inflammatory monocytes/neutrophils and immunoregulatory lymphocytes. Elevated SIRI indicates a shift toward a pro-tumor inflammatory environment and increased cancer risk. Key findings from this study demonstrate that elevated SIRI is a risk factor for invasive breast cancer (IBC) (OR = 1.906, P = 0.002), meaning high SIRI patients face a 90.6% higher breast cancer risk. Additionally, Zhu M et al. found that lower SIRI scores predict longer overall survival and disease-free survival in post-surgery breast cancer patients (23); a meta-analysis of 2, 997 breast cancer patients further confirmed that high SIRI correlates with T3-T4 stage (OR = 1.73, 95% CI = 1.40-2.14, p<0.001), N1-N3 stage (OR = 1.61, 95% CI = 1.37-1.91, p<0.001), TNM stage III (OR = 1.63, 95% CI = 1.34-1.98, p<0.001) and poorly differentiated tumors (OR = 1.25, 95% CI = 1.02-1.52, p=0.028) (24). The predictive value of SIRI for breast cancer can be explained by the stimulatory role of inflammatory cells in tumor initiation and progression.

Neutrophils, the most abundant circulating leukocytes, exert dual pro-tumor and anti-tumor effects in breast cancer pathogenesis, with their functions regulated by the tumor microenvironment (TME). Core mechanisms include tumor-associated neutrophil (TAN) phenotypic polarization, neutrophil extracellular trap (NET) formation and immune suppression, all closely linked to tumor progression, metastasis and drug resistance (25). TGF-β in the TME induces TAN polarization to the pro-tumor N2 phenotype, which suppresses cytotoxic T and NK cell function via cathepsin-1 and reactive oxygen species (ROS), and promotes angiogenesis and tumor invasion by secreting VEGF and MMPs (26, 27). Conversely, type I interferons drive TAN polarization to the anti-tumor N1 phenotype, which secretes TNF-α to recruit effector immune cells (28).

Additionally, NETs (DNA-rich structures released by neutrophils) trap circulating tumor cells and mediate their interactions with platelets and endothelial cells, facilitating distant organ seeding (29). In triple-negative breast cancer (TNBC), NETs also contribute to pre-metastatic niche formation and enhance metastatic potential (30). Neutrophils further mediate breast cancer treatment resistance by regulating cancer stem cell function and impairing targeted therapy efficacy. Neutrophil-secreted IL-6 and IL-8 enhance breast cancer stem cell survival and self-renewal, driving tumor recurrence and resistance (26). In HER2-positive breast cancer, TAN-derived ROS impairs NK cell function, reduces trastuzumab-mediated antibody-dependent cellular cytotoxicity (ADCC) and induces trastuzumab resistance (31).

Lymphocytes exert critical tumor-suppressive effects by inducing cytotoxic cell death and secreting cytokines that inhibit cancer cell growth and metastasis. Tumor-infiltrating lymphocytes are considered a favorable prognostic marker (32). In colorectal cancer, memory T cells modulate the tumor microenvironment or tumor cells during adaptive immune responses to reduce metastatic potential, and the trafficking characteristics, density and sustained antitumor capacity of T cells are central to controlling tumor recurrence (33). Consistently, tumor-infiltrating lymphocytes in solid tumors exhibit oligoclonal proliferation, tumor antigen recognition and tumor-specific cytotoxic activity *in vitro*, contributing to improved clinical outcomes including delayed recurrence and reduced mortality. Therefore, elevated SIRI levels resulting from lymphopenia and/or neutrophilia may indicate impaired antitumor immune responses and increased risk of tumor recurrence.

Monocytes, core regulatory cells in the breast cancer tumor microenvironment (TME), are deeply involved in tumorigenesis, progression and metastasis via expansion and differentiation into functional subtypes. Clinical studies comparing breast cancer patients with healthy controls showed significantly higher CD14^+^CD16^+^ monocyte levels in patients, which were closely associated with early-stage breast cancer (Stage I, T1-T2 tumors) but not with lymph node metastasis, hormone receptor (ER/PR) status or HER2 expression (34). Laboratory studies further demonstrated that conditioned medium from MCF-7 breast cancer cells (MCF-CM) induced dose-dependent expansion of CD14^+^CD16^+^ monocytes: 25% MCF-CM caused a 3-fold increase, while 50%-75% MCF-CM resulted in a 5-fold increase (34). Mechanistically, monocytes suppress T cell proliferation via PD-L1, IL-10 and TGF-β, and impair T cell function by producing NO through arginase-1 (ARG1) and iNOS, thereby promoting breast cancer development (35). They also accelerate tumor angiogenesis by secreting VEGF, PDGF and IL-8, degrade the extracellular matrix (ECM) via MMP1/2/9 and cathepsins, and facilitate tumor cell metastasis through the CSF-1/EGF paracrine loop (36).

This study examined associations between inflammation, metabolism, nutrition, and IBC risk, identifying MS, PNI, and SIRI as independent risk factors for breast cancer development with good representativeness and clinical guidance value. However, several limitations of this study should be noted. First, this single-center retrospective study conducted at a tertiary hospital in northern China may have inherent selection bias. All participants were surgical patients with breast masses, so the findings cannot be extrapolated to asymptomatic community populations or non-surgical patients, limiting generalizability.

Second, although we identified independent risk factors for invasive breast cancer (IBC) via multivariate logistic regression, no clinically applicable prediction model (e.g., risk score, nomogram) was developed, restricting the translational value of our findings for routine practice. Future studies should integrate these factors into a validated model for individualized breast cancer risk stratification. Third, key oncological variables including tumor stage, histological grade, and molecular subtype (ER, PR, HER2 status) were not included. While our study focused on pre-diagnostic risk factors rather than prognostic markers, these variables may interact with inflammatory, nutritional, and metabolic indicators, potentially confounding the observed associations. Future research should explore these relationships across different molecular subtypes. Fourth, non-specific inflammatory and nutritional markers (e.g., SIRI, PNI) may be affected by acute and chronic conditions such as infections and autoimmune diseases. Although we excluded patients with concurrent malignancies and incomplete records, we did not systematically adjust for these confounders, which may have weakened the validity of our conclusions. Future prospective studies should collect detailed comorbidity data for more comprehensive adjustment.

In conclusion, advanced age, postmenopausal status, metabolic syndrome, reduced preoperative nutritional index, and elevated systemic inflammatory response index are independent risk factors for invasive breast cancer in women. A history of benign breast surgery may represent a potential protective factor against invasive breast cancer in women. Clinicians can develop individualized screening strategies targeting these high-risk factors, such as enhancing breast monitoring for elderly, postmenopausal women with metabolic syndrome, and incorporating PNI and SIRI assessments to evaluate risk. This approach facilitates early diagnosis and intervention for invasive breast cancer, thereby improving patient prognosis.

## Data Availability

The original contributions presented in the study are included in the article/supplementary material. Further inquiries can be directed to the corresponding authors.
